# Population-level, patient-reported outcomes: a case study regarding a public health intervention that involves patients with life-limiting illnesses

**DOI:** 10.3389/fpubh.2023.1232881

**Published:** 2023-08-10

**Authors:** Barbara Daveson, Megan Blanchard, Sabina Clapham, Kylie Draper, Alanna Connolly, David Currow

**Affiliations:** ^1^Palliative Care Outcomes Collaboration, University of Wollongong, Wollongong, NSW, Australia; ^2^Faculty of Science, Medicine and Health, University of Wollongong, Wollongong, NSW, Australia

**Keywords:** palliative care, aged care, health status disparities, public health, mixed methods

## Abstract

**Introduction:**

Dying and death are public health concerns, but little is known about public health interventions that target populations living with life-limiting illnesses. This gap makes it difficult to identify best-practice public health interventions for this population and to achieve public health objectives. The study aimed to describe a public health intervention that intends to improve population-level outcomes using point-of-care and patient-reported outcomes.

**Methods:**

A case study approach, informed by the Organization for Economic Co-operation and Development's (OECD) Best-Practice Public Health Framework, was used to describe coverage, effectiveness, and equity using mixed methods. Data from 2012 to 2022 were analyzed.

**Results:**

Over the 10-year period, the number of deaths recorded in the programme (*n* = 16,358 to 32,421, +98.2%) as well as the percentage of the population that might benefit from palliative care increased (14.8% to 25.1%). The median age of those admitted for care (74 to 77 years) and the proportion of services participating in the programme located in outer regional and remote areas of Australia increased (2012: 59; 2022: 94; +5.4%). The access by patients that experience the greatest socioeconomic disadvantage decreased (2012: 18.2% *n* = 4,918; 2022: 15.9% *n* = 9,525). Improvements in relation to moderate distress related to pain were identified (2012: 63% *n* = 8,751, 2022: 69% *n* = 13,700), and one in five instances of severe distress related to pain did not improve (2012: 20% *n* = 781; 2022: 19% *n* = 635).

**Conclusion:**

Population-level, patient-reported outcome data are useful and necessary in addressing public health objectives in populations with life-limiting illnesses. Our application of the OECD's Best-Practice Public Health Framework has helped to identify and describe a national intervention that may be transferred to other settings to address health promotion objectives. This may help improve the targeting of treatments to improve pain and issues related to equity.

## Introduction

Public health and palliative care have much in common. Both disciplines include a commitment to achieving the optimal health for everyone in society and rely on data and evidence-informed strategies to achieve this aim (1, 2). Population-level data are also key to advancing both disciplines.

Population-level data, or big data, provide valuable insights into public health objectives. They can assist with health planning, the prediction of risk (including risks related to under-served populations), the targeting of interventions, the understanding of disease (including its trajectory), and issues related to safety and harm (3). Examples of the use of big data within palliative care include the use of national death indices to estimate populations (4), hospital activity information to derive risks (5), government-subsidized pharmaceutical dispensing data to illuminate inequities regarding palliative-care-related medicines (6), surveys to investigate bereavement needs (7), differential impacts on caregivers when specialist palliative care services are and are not accessed (8), and the needs of caregivers who did or did not use a specialist palliative care service (9).

However, population-level data that report on patient-reported outcome measures that closely align with clinical care are rarely available. This gap hinders public health evaluation of populations with life-limiting illnesses and the monitoring of the effectiveness of health systems. Populations with life-limiting illnesses often require the use of a specialized, multidisciplinary care in which the primary aim is to optimize the person's functioning (to maintain independence for as long as possible) and quality of life. Routinely collected clinical data, which capture information about symptom burden, performance, and clinical acuity are therefore useful for promoting the health of populations at the end of life (3). Patient-reported outcome indicators ensure that the users of the health systems (i.e., the patients themselves) can directly influence the evaluation of the health systems that they use. There is a growing recognition of the need to incorporate patient experience measures (e.g., ease of access to information) and patient preferences (e.g., place of care) but less recognition of the need to report on patient-reported outcomes, including the extent of distress that patients may experience.

Close partnerships between clinician communities, patients and their caregivers, and key groups, such as universities, are likely to be key to the successful development and adoption of public health interventions in palliative care (10). Despite this, the focus of partnerships in public health has often included an emphasis on partnerships with community organizations and faith-based groups, as well as members of the public (11). Less emphasis has been placed on partnerships from within the professional healthcare system (e.g., communities of practices inclusive of palliative care service providers), and the involvement of patients, their caregivers, and the public is also often neglected. Typically, the involvement of patients, caregivers, and the public has evolved by including them as the target audience of public health initiatives rather than as partners that can help shape and inform the programme itself (11).

Whilst the recognition of the need for public health for populations with life-limiting illnesses is growing, the descriptions of population-based, public health interventions are lacking. Describing public health interventions is a critical first step to identifying public health interventions that may be useful for national implementation (2). This study aims to assess a national initiative that intends to improve population-level outcomes for people with life-limiting illnesses. The initiative is called the Australian National Palliative Care Outcomes Collaboration (PCOC) (12). PCOC has previously been demonstrated as feasible, desirable, and useful in addressing public health objectives (12, 13) although scant accounts with respect to describing how PCOC may explicitly align with the available public health methodology. This study aims to help address this gap in knowledge.

## Methods

We used the Organization for Economic Co-operation and Development (OECD) Best-Practice Public Health Intervention Framework to assess the PCOC intervention (2). A case study approach was used to inform the evaluation of the PCOC intervention in line with the OECD 5E Framework. The areas assessed included the extent of coverage, effectiveness, and equity (2). Descriptive statistics were used to describe changes over time to compare the proportions in 2012 and 2022, and the percentage increases or decreases between these two points.

### Extent of coverage

Coverage was assessed using two measures. Change was calculated for the period 2012–2022 regarding the volume of services registered with the programme and in relation to a range of service characteristics. This was to be presented as the total number of services divided by care setting (inpatient and community), service size, and location. The size of the service was derived by examining episodes of care in each service, with an episode of care defined as a continuous period of care for a patient in one setting.

The second measure involved the use of a well-established method of estimating the need for palliative care, developed by Murtagh et al. (14). This measure was used to provide an indication of the extent of coverage of the intervention over time in relation to the estimated need within the total population. This methodology was selected for use due to its expanded inclusion of ICD-10 codes and its more comprehensive consideration of underlying and contributory causes of death and inpatient admission patterns prior to death as compared to other methodologies (15, 16).

For the analysis, the deaths recorded in the PCOC programme were calculated as a proportion of people who could potentially benefit from palliative care and analysis of this with the Australian Bureau of Statistics Cause of Death Data from 2012 to 2021. The estimate included using the number of people with a selected underlying cause of death plus a contributing cause of death for selected conditions (to estimate co-morbidities). The conditions included were all-cancer deaths (C00-C97—malignant neoplasms only included) and selected non-cancer deaths (*ICD-10*: I00-I52, I60-I69, N17, N18, N28, K70-K77, J06-J18, J20-J22, J40-J47 and J96, G10, G20, G35, G122, G903, G231, F01, F03, G30, R54, B20-B24) (14). The most recent and complete 10-year period was included in the analysis, that is, from January 2012 to December 2021. As 2022 data were not available at the time of our analysis, we derived an estimate based on data from previous years.

### Effectiveness

In relation to effectiveness, improvements in a key symptom area were examined to describe population-level changes, that is, pain. The OECD's expert guidance is that intervention-specific health indicators can be used to assess effectiveness if there is a need to assess the extent to which an intervention's desired outcomes were achieved in a real-world setting. The trends in the ways in which distress related to pain was managed over the 10-year period were examined. Distress was measured using the patient-reported PCOC Symptom Assessment Scale (PCOC SAS) (17), which is a derivative of earlier scales (18–21). PCOC SAS is an 11-point numerical rating scale with the response options on the scale grouped into six categories. Each category has a corresponding descriptor, color, and facial expression for assisting the patient in reporting their distress. Higher scores represent higher levels of distress. Descriptive statistics were used to describe a 1-point change in the 11-point scale. Instances of positive, negative, or no change from scores were also derived. This was calculated by using the scores that varied from the absent (a score of 0) to mild (scores 1–3), moderate (scores 4–7), and severe (scores 8–10) ranges of the scale.

### Equity

In relation to equity, a measure of socioeconomic disadvantage was calculated for each patient that accessed care over the 10-year period. This was completed to report trends by the socioeconomic disadvantage. The measure we used is the Socio-Economic Indexes for Areas (SEIFA) quintiles (22), which are based on the ABS Index of Relative Socio-Economic Disadvantage. Each SEIFA quintile represents ~20% of the national population, with quintile one being the most disadvantaged and quintile five being the most advantaged.

## Results

### Extent of coverage

The extent of coverage of the programme increased in relation to palliative care services registered, patient outcomes captured, the proportion of patients in Australia that may benefit from palliative care, and the proportion of those that die in Australia.

In relation to the coverage of palliative care services, the absolute number of palliative care services registered with the programme increased from 135 to 215 services (+59%). An increase in the proportion of larger services (i.e., 300+ episodes of care per 6 months) was observed, primarily including an increase in the growth in larger community services in the country (Table 1).

**Table 1:** Characteristics of palliative care services registered with the PCOC programme in 2012, 2022, and the percentage increase and decrease observed.

**Characteristics**	**2012**	**2022**	**Percentage increase or decrease**
	***n*** **(%)**	***n*** **(%)**	
**Services**			
Number of services	135	215	+59.3%
**Location of service**			
Major city	76 (56.3)	121 (56.3)	+0.0%
Inner regional	43 (31.9)	57 (26.5)	−5.3%
Outer regional/remote	16 (11.9)	37 (17.2)	+5.4%
**Care setting**			
Inpatient	81 (60.0)	129 (60.0)	0.0%
Community	54 (40.0)	86 (40.0)	0.0%
**Size of service**			
Small (< 100 episodes)	47 (34.8)	75 (34.9)	+0.1%
Medium (100–299 episodes)	44 (32.6)	65 (30.2)	−2.4%
Large (300+ episodes)	44 (32.6)	75 (34.9)	+2.3%
**Care setting by the size of service**			
**Inpatient**			
Small (< 100 episodes)	26 (19.3)	47 (21.9)	+2.6%
Medium (100–299 episodes)	24 (17.8)	38 (17.7)	−0.1%
Large (300+ episodes)	31 (23.0)	44 (20.5)	−2.5%
**Community**			
Small (< 100 episodes)	21 (15.6)	28 (13.0)	−2.5%
Medium (100–299 episodes)	20 (14.8)	27 (12.6)	−2.3%
Large (300+ episodes)	13 (9.6)	31 (14.4)	+4.8%

In relation to the extent of coverage of patients, the absolute number of patients in the programme increased by 110% (*n* = 28,528 annually to *n* = 60,032 annually), and the overall median age of patients observed by the services increased from 74 years to 77 years. An increase in the proportion of patients diagnosed with a principal life-limiting illness other than cancer, a decrease in the proportion of pediatric patients, and an increase in the proportion of adolescents, young adults, and older adults (+85 years) were evident (Table 2).

**Table 2:** Characteristics of palliative care patients admitted to services registered with the PCOC programme in 2012, 2022, and the percentage increase and decrease observed.

**Characteristics**	**2012**	**2022**	**Percentage increase or decrease**
	***n*** **(%)**	***n*** **(%)**	
**Age group**			
≤ 15	120 (0.3)	62 (0.1)	−0.2%
16–25	1,083 (2.9)	3,773 (4.8)	+1.9%
26–39	552 (1.5)	1,132 (1.4)	0.0%
40–64	9,210 (24.8)	14,332 (18.2)	−6.6%
65–79	14,132 (38.1)	27,325 (34.7)	−3.4%
≥80	12,036 (32.4)	32,121 (40.8)	+8.4%
Median age in years (IQR)	74 (63–82)	77 (67–85)	
**Sex**			
Men	15,202 (53.3)	31,191 (52.0)	−1.3%
Women	13,297 (46.7)	28,778 (48.0)	+1.3%
Not stated/other	29	63	
**Country of birth**			
Australia	17,885 (64.9)	37,755 (64.5)	+0.4%
Not Australia	9,663 (35.1)	20,787 (35.5)	−0.4%
**Preferred language**			
English	13,871 (88.9)	52,900 (89.4)	+0.5%
Other than English	1,739 (11.1)	6,275 (10.6)	−0.5%
**Primary diagnosis**			
Cancer	22,654 (80.6)	36,942 (63.2)	−17.5%
End-stage organ failure	2,645 (9.4)	8,770 (15.0)	+5.6%
Other non-cancers	1,834 (6.5)	6,541 (11.2)	+4.7%
Neurodegenerative disease	946 (3.4)	3,117 (5.3)	+2.0%
Alzheimer's Disease and other dementias	17 (0.1)	3,121 (5.3)	+5.3%
**SEIFA—IRSAD quintile**			
1 (greater disadvantage)	4,918 (18.2)	9,525 (15.9)	−2.3%
2	3,769 (14.0)	8,907 (14.9)	+0.9%
3	4,721 (17.5)	11,450 (19.1)	+1.6%
4	4,645 (17.2)	12,404 (20.7)	+3.5%
5 (greater advantage)	8,913 (33.1)	17,606 (29.4)	−3.7%

**Figure 1:**
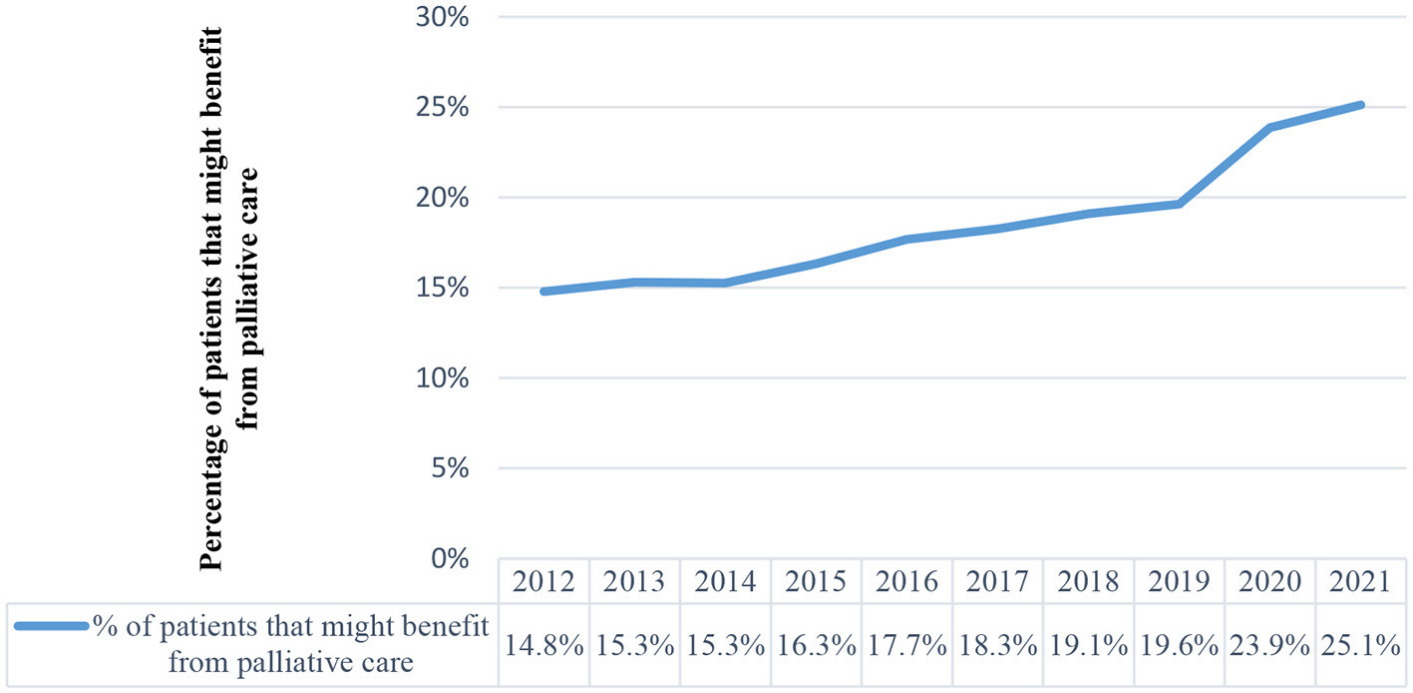
Proportion of patients in Australia that may benefit from palliative care and the proportion of deaths in Australia reported in the national programme.

The number of deaths reported in PCOC increased each year both in absolute terms and as a percentage of patients who might potentially benefit from palliative care (14.8% to 25.1%). In 2012, the national initiative reported 16,358 deaths, which increased to 32,421 deaths in 2022 (+98.2%) (Figure 1).

### Effectiveness

**Figure 2:**
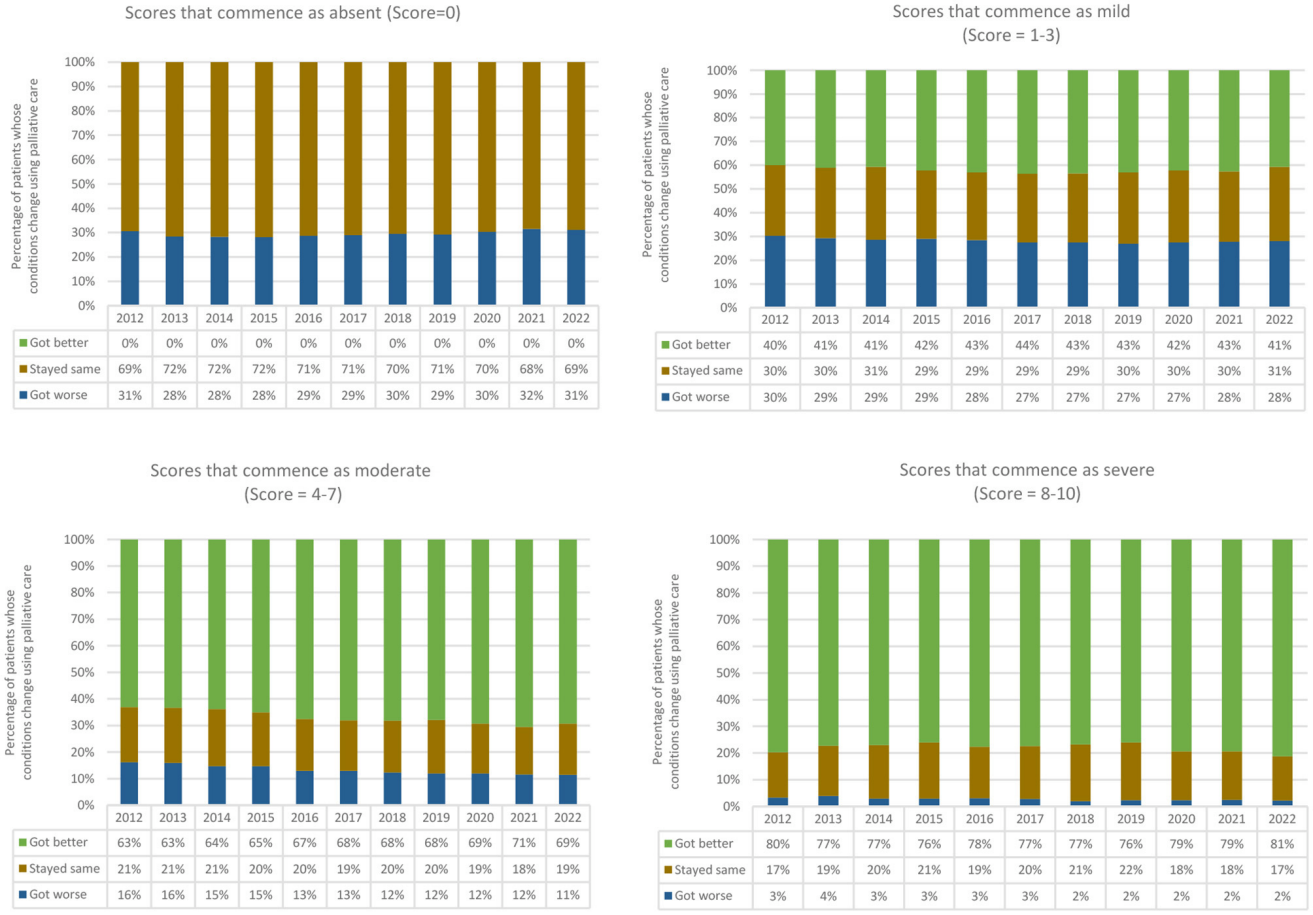
Proportion of change in distress related to pain over a 10-year period by absent, mild, moderate, and severe scores.

In relation to distress related to pain (as measured by the PCOC SAS), the trends remained broadly the same over the 10-year period (Table 3). Further examination identified substantial improvements in relation to moderate distress related to pain over the same period. An increase in the proportion that improved was observed (63% to 69%), whilst the proportion that got worse decreased (16% to 11%). In relation to absent and mild scores, the proportion of outcomes (that got worse, stayed the same, or improved) remained constant. One in five reports of severe distress stayed the same or got worse over the 10-year period (18% in 2012 and 20% in 2022) (Figure 2).

**Table 3:** Number and proportion of patient outcomes that worsened, stayed the same, or improved over time: distress related to pain.

	**Distress related to pain (PCOC SAS)**					
**Year**	**Worsened** ***n*** **(%)**		**Stayed the same** ***n*** **(%)**		**Improved** ***n*** **(%)**	
2012	7,558	(22%)	8,626	(25%)	18,565	(53%)
2013	9,633	(22%)	11,264	(25%)	23,797	(53%)
2014	10,616	(21%)	13,232	(26%)	26,554	(53%)
2015	11,692	(21%)	13,645	(25%)	29,440	(54%)
2016	12,139	(21%)	14,290	(25%)	31,484	(54%)
2017	12,279	(21%)	14,716	(25%)	31,637	(54%)
2018	13,131	(21%)	16,039	(26%)	33,354	(53%)
2019	14,181	(21%)	18,025	(26%)	35,875	(53%)
2020	16,317	(21%)	19,981	(26%)	39,716	(52%)
2021	16,770	(22%)	19,635	(26%)	39,862	(52%)
2022	16,503	(22%)	20,222	(27%)	37,008	(50%)

### Equity

The proportion of services participating in the programme located in outer regional and remote areas of Australia increased by +5.4% (Table 1). A substantial decrease in the proportion of patients that experience the greatest disadvantage within Australia was observed. This group was under-represented in 2012 (18.2%) and even more so by 2022 (15.9%). The proportion of patients in the services within the top quintile reduced from 33.4% to 29.4% (Table 2).

## Discussion

The public recognizes the importance of dying and death; they are often concerned about any perceived prioritization of the quantity of life over the quality of life with respect to people with life-limiting illnesses; and they call for improved quality of end-of-life care and palliative care for patients, especially for older adult populations and their carers (e.g., families and friends) (23). Given these public priorities, our study provides useful insights into a public health concern as we present effectiveness, coverage, and equity data from a national initiative focused on those with life-limiting illnesses. Improved coverage of the population living with life-limiting illnesses within the national programme was evident over the 10-year period although trends revealed that those that experience the greatest disadvantage within Australia are the ones less likely to be admitted to care. Our analysis of data from the national initiative has also shown that gains have been made in relation to the health of those living with moderate distress related to pain; however, there is little evidence of improved resolution of severe distress related to pain. An unexpected finding was the increase in the median age of patients admitted to care, which may be explained by the increase in life expectancy within Australia. Life expectancy increased by 0.9 years [from 82.3 years (24) to 83.2 years (25)] over the 10-year period studied in the study, with the volume of the highest annual increase in the population growth of the 75 to 84-year-old age group estimated to peak in the early years of 2020 (26). The increase in the median age of patients accessing care may be explained by this growth within society (25).

Our study also showed that the number of deaths reported in PCOC increased each year both in absolute terms and as a percentage of patients who might potentially benefit from palliative care (14.8% to 25.1%). In 2012, the national initiative reported 16,358 deaths, which increased to 32,421 deaths in 2022 (+98.2%). Whilst this coverage represents a major achievement by the national voluntary initiative, it is important to emphasize that the methodology we used to estimate the need for palliative care relies on the assumption that people who are missing out on accessing palliative care have unmet needs (14, 15, 27). Whilst this methodology has been useful, the assumption underpinning the model has limitations (9). The limitations include how it fails to account for the effectiveness of other providers of care (e.g., primary palliative care) and patient preference. The range of methodologies that were available for us to estimate the need for palliative care for our study all failed to address these underlying assumptions. Higginson et al.'s disease-specific methodology includes a range of cancer diagnoses and six non-cancer diagnoses, with the consideration of symptom prevalence (14). Rosenwax et al.'s method relies on routine mortality statistics to estimate the need for palliative care for cancerous and non-cancerous populations, using all deaths from 10 specific disease groups (16). Gómez-Batiste et al.'s methodology is informed by the estimated proportion of deaths from chronic progressive diseases and its prevalence (15). Whilst we selected Murtagh et al.'s method because of its expanded inclusion of ICD-10 codes and its more comprehensive consideration of underlying and contributory causes of death and inpatient admission patterns prior to death, it is important to discuss these assumptions.

An alternative approach that we could have used involves the recognition that a referral to a service may not necessarily equate with a need and that unmet or perceived needs may not necessarily equate to a referral or a preference to be referred (9). This means that a lack of admission to a palliative care service may not equate to an unmet need. As described elsewhere, an alternative approach that incorporates this alternative view could allow for the identification of a group that had used a service and benefited from it (e.g., primary palliative care ± specialist palliative care), a group that had used a service but not benefited from the service (e.g., primary palliative care or specialist palliative care, or a combination of both), a group in the population where a service was not used (e.g., primary or specialist palliative care) but it would not have added value, and a group where a service was not used (e.g., as it was not available or the patient preferred not to use the service) but yet the service may have added benefit (e.g., primary or specialist palliative care, or a combination of both) (9, 28). The continued surveillance of the accessibility of palliative care services can allow for a population-based gap analysis to be completed, especially as the coverage of PCOC in primary palliative care expands. Continued growth in the programme may allow for the analysis of those that access specialist palliative care and/or primary care (with or without primary palliative care) and changes in outcomes of these groups. One of the original aspirations of the PCOC programme was to improve outcomes at scale, and this also includes the monitoring of outcomes across the country (12). At present, the PCOC programme can begin to achieve this goal as the PCOC dataset has matured. This development was also anticipated by the founders of the programme as early as 2008 (12).

A key objective of public health is to ensure the promotion of health for all in society and not just those that can afford to access care or the majority within society. Our study reports trends related to the socioeconomic disadvantage indicative of growing inequities in relation to service entry. It suggests that resource use by patients continues to be inequitable (assuming preferences to access the service are constant across the quintiles) and that this disparity is increasing. This is because 20% of the population with a greater economic disadvantage within Australia are less likely to access palliative care. Inequities in relation to accessing specialist palliative care in Australia based on its geography have been previously described in a study that geocoded palliative care services nationally (using postcode) to one nationally standardized measure of socioeconomic deprivation and the location of the inpatient service and each person's home postcode. The earlier study showed that, on average, those that were least socioeconomically disadvantaged had to travel 14 km to their closest inpatient palliative care service, whilst those that were most disadvantaged had to travel three times the distance to be able to receive inpatient palliative care. This earlier study also analyzed PCOC data (29).

The intervention we examined in our study involved a close partnership between a community of practice that involves clinical services and a university (10). Our case study, therefore, adds valuable information to supplement a gap in the literature that places an emphasis on the involvement of groups outside of the professional healthcare system in relation to public health interventions (11). A relevant limitation for PCOC though is the lack of any description of how PCOC engages with patients, caregivers, and the public to help develop and inform the programme. This means that PCOC, similar to other initiatives, has an opportunity to engage more fully with members of the public as partners to help shape and inform the development of the programme. Involving the public in the ongoing development of this initiative may help derive ways to expand the coverage of the programme, develop measures of unmet needs, and help support population-based planning (30).

### Strengths and limitations

Changes in population structures, diseases, and risk factors (e.g., lifestyle behaviors) have led to growing public health challenges. In response, policymakers are experimenting with different interventions that improve population health in a sustainable way. However, achieving public health objectives continues to be challenging. One of the strengths of our study is the use of the OECD's 5E Framework because the framework has provided a pragmatic approach to begin to identify and evaluate a public health intervention that may be transferrable to other countries and settings, and in doing so, we have addressed the dearth of population-level public health interventions that focus on those with life-limiting illnesses (2). A second strength is the use of a patient-reported measure to help evaluate health system performance. These types of measures are rarely implemented nationally, and therefore, they are rarely available to assist with health systems monitoring. However, limitations of our application of the framework include the lack of a more comprehensive evaluation of the effectiveness of the public health intervention, alongside an examination of its efficiency, and the evidence-based one used to inform the programme. These areas should be addressed. Our study also fails to account for growth in the development of palliative care services (and therefore improved availability) throughout Australia. Nevertheless, future evaluations regarding these dimensions are possible, especially as the PCOC programme is being implemented within a range of other countries. As discussed earlier, a key limitation of our study rests with its reliance on methods to derive estimates of needs that equate access to care with unmet needs and the lack of data regarding primary palliative care.

## Conclusion

Public health and palliative care have much in common. Both disciplines include a commitment to optimal health for all and the use of data to achieve this aim. Population-level, patient-reported outcome data are useful and necessary in addressing public health objectives in populations with life-limiting illnesses. Our application of the OECD's Best-Practice Public Health Framework has helped identify a national intervention that may be transferred to other settings to address health promotion objectives, especially in relation to the effective targeting of treatments and issues related to equity.

## Data availability statement

The original contributions presented in the study are included in the article/supplementary material, further inquiries can be directed to the corresponding author.

## Ethics statement

The studies involving human participants were reviewed and approved by University of Wollongong and Illawarra Shoalhaven Local Health District Health and Medical Human Research Ethics Committee (2021/ETH00988). Written informed consent from the participants' legal guardian/next of kin was not required to participate in this study in accordance with the national legislation and the institutional requirements.

## Author contributions

BD conceived and led the drafting of the article. All other authors helped refine and develop the concept of the article, collect data, refine the material analyzed and presented and the methods used for this study and analysis, and they also developed an interpretation of the findings.
